# The Impact of CFTR Modulator Triple Therapy on Type 2 Inflammatory Response in Patients with Cystic Fibrosis

**DOI:** 10.21203/rs.3.rs-2846739/v1

**Published:** 2023-05-10

**Authors:** Ajay Mehta, Irene Lee, Galvin Li, Marieke Jones, Lydia Hanson, Kevin Lonabaugh, Rhonda List, Larry Borish, Dana Albon

**Affiliations:** University of Virginia School of Medicine; Baylor College of Medicine; University of Virginia School of Medicine; University of Virginia Department of Public Health Sciences; University of Virginia School of Medicine; University of Virginia School of Medicine; University of Virginia School of Medicine; University of Virginia School of Medicine; University of Virginia

## Abstract

**Background:**

Treatment of cystic fibrosis (CF) has been revolutionized by the use of cystic fibrosis transmembrane conductance regulator (CFTR) protein modulators such as elexacaftor/tezacaftor/ivacaftor (ETI) triple therapy. Prior studies support a role for type 2 (T2) inflammation in many people with CF (PwCF) and CF-asthma overlap syndrome (CFAOS) is considered a separate clinical entity. It is unknown whether initiation of ETI therapy impacts T2 inflammation in PwCF. We hypothesized that ETI initiation decreases T2 inflammation in PwCF.

**Methods:**

A single center retrospective chart review was conducted for adult PwCF. As markers of T2 inflammation, absolute eosinophil count (AEC) and total immunoglobulin E (IgE) data were collected longitudinally 12 months prior to ETI therapy initiation and 12 months following therapy initiation. Multivariable analyses adjusted for the age, gender, CFTR mutation, disease severity, inhaled steroid use, and microbiological colonization.

**Results:**

There was a statistically significant reduction (20.10%, p < 0.001) in 12-month mean IgE following ETI initiation; this change remained statistically significant in the multivariate model. The longitudinal analysis demonstrated no change in AEC following therapy initiation.

**Conclusion:**

This study shows reduction in IgE but no change in AEC after ETI therapy initiation. We think that the lack of influence on AEC argues against an impact on previously established T2 inflammation and that the reduction in IgE is likely related to antigen load reduction post ETI. Further studies are warranted to determine the underlying mechanism of ETI impact on T2 inflammation and possible role for asthma immunomodulator therapy post ETI initiation in CFAOS.

## Introduction

Cystic fibrosis (CF) is one of the most common respiratory genetic diseases, affecting an estimated ~37,000 people in North America and ~162,000 people worldwide^1^. It is characterized by mutations in the *CFTR* gene which encodes the cystic fibrosis transmembrane conductance regulator (CFTR) protein, most commonly a deleted phenylalanine in the 508^th^ position (F508del). CFTR is a chloride channel that regulates transport of sodium, chloride, and bicarbonate.^2^ Classic pulmonary symptoms of CF include thick, mucous secretions in the airway causing chronic pulmonary infection, increase bacterial load and fungal colonization, and obstructive lung symptoms, which progress to airway edema, mucus plugging with airway collapse, and bronchiectasis.^2^

Many studies have shown that CF patients commonly display evidence of type 2 (T2) inflammation^3^ characterized by evidence of airway and peripheral eosinophilia, as well as high levels of total and allergen-specific immunoglobulin E (IgE).^3,4^ T2 inflammation is classically implicated in the pathogenesis of allergic as well as non-allergic eosinophilic subtypes of asthma.^3,5^ but can also develop in other lung inflammatory disorders including, for example, being highly prevalent in eosinophilic chronic obstruction pulmonary disease. Similar epithelial differentiation can be conjectured as often evolving in the inflamed airway of CF patients. In these scenarios, T2 inflammation represents, in large part, the response of the airway epithelial cells to airspace inflammation with the consequent production of numerous cytokines (including the alarmins thymic stromal lymphopoietin (TSLP), interleukin (IL)-25, and IL-33) and chemokines (including CCL5, CCL11, CCL24, CCL26) that are associated with promoting the T2 inflammation signature. These mediators both directly drive the generation of the classic T2 cytokines IL-4, IL-5, and IL-13 from innate lymphoid 2 cells (ILC2s), mast cells, and others but are also responsible for the differentiation of the antigen-specific Th2 effector cells that also produce these cytokines. Loss of epithelial barriers allow for allergens and microbes to access stromal tissue and further promote the activation of Th2 effector lymphocytes and B cells.^5^ Together these cytokines underlie the presence of both eosinophilic inflammation (IL-5) and elevated concentrations of IgE (IL-4 and IL-13).

Several studies support this role for T2 inflammation in many patients with CF. Siedlecki, *et. al.* found a prevalence of peripheral eosinophilia of 45% in CF patients hospitalized for exacerbation, and eosinophilia was associated with significantly longer hospital stays.^6^ Our group previously found a correlation between T2 inflammation, frequent exacerbations and lung function decline, based on peripheral eosinophilia and IgE levels and we have reported on the ability of eosinophil-targeting therapeutics to produce beneficial outcomes in CF patients.^7,8^ Interestingly, according to the 2021 CF Foundation Patient Registry Annual Data Report, the prevalence of asthma in CF patients is 30.8 %.^9^ Experts in the CF community have acknowledged the association between asthma and CF, now considering CF-asthma overlap syndrome (CFAOS) a separate clinical entity.^10^ Previous studies support this concept that elevations in type 2 inflammatory markers likely reflect an intrinsic eosinophilic phenotype of CF.^4,5^

Elexacaftor/tezacaftor/ivacaftor (ETI) modulator therapy has recently been approved by Food and Drug Administration and has become the main therapy prescribed for most people with CF. ETI has been shown to improve CFTR function, decrease sweat chloride concentrations, and improve both lung function and functional status as demonstrated in the PROMISE clinical trial^11^. While addressing the defect in CFTR function, it is unknown whether these agents impact the presence of a previously established T2 inflammatory state that developed as a complication of CF inflammation.

The aim of this study was to investigate the impact of CFTR modulator triple therapy on Absolute Eosinophil Count (AEC) and IgE as markers of the type 2 inflammatory response in patients with CF.

## Methods

### Patient Population

A single center retrospective chart review was conducted for adult cystic fibrosis patients (n = 108) seen at the University of Virginia Adult Cystic Fibrosis Clinic between 2018 and 2021. Inclusion criteria were a diagnosis of CF and initiation of ETI therapy. Exclusion criteria were a history of transplant, IL-5 (benralizumab, mepolizumab) or IgE (omalizumab)-targeting biologics, and insufficient AEC or IgE data. Following exclusion, there were 54 patients with adequate AEC data and 80 patients with adequate IgE data for a total of 85 patients. This study was approved by our institution’s Institutional Review Board Human Subjects Research committee and followed procedures with ethical standards. (IRB-HSR 23337).

### Data Collection

AEC and IgE data were collected for each patient 12 months prior to initiation of ETI therapy and 12 months following initiation of therapy. AEC measurements from the center-associated lab were collected from the electronic health record (EHR). Given that infection results in activation of inflammatory responses, we excluded data collected during pulmonary exacerbation and/or use of IV antibiotics. AEC measurements were excluded if they were obtained within 7 days prior to determination of an exacerbation and/or initiation of IV antibiotic therapy or if they were within 14 days following exacerbation and/or completion of IV antibiotic therapy.

We were unable to exclude IgE measurements taken during an exacerbation as IgE measurements were obtained on a less frequent basis, often only once annually. As such, IgE data were collected from the EHR including both data from the center associated lab and outside facilities, where available. Additional data collected included age, sex, disease severity, infection/colonization history, CFTR mutation information, and inhaled steroid use. Disease severity was defined based on percent predicted Forced Expiratory Volume in one second (ppFEV_1_) and was categorized as normal (ppFEV_1_ > 90%), mild (70% <ppFEV_1_ ≤ 90%), moderate (40% <ppFEV_1_ ≤ 70%), and severe (ppFEV_1_ ≤ 40%). With regards to bacterial infection/colonization history, data regarding prior *Pseudomonas* infection, MRSA infection, and other bacterial colonization were collected. Other bacteria were defined as *Stenotrophomonas* spp., *Achromobacter* spp., *Acinetobacter* spp., or *Burkholderia* spp. With regards to fungal colonization history, data regarding prior *Aspergillus* spp., *Exophiala* spp., and *Rasamsonia* spp. colonization were collected. Inhaled steroid use was coded as yes or no, defined as use prior to ETI initiation.

### Statistical Analysis

All statistical analyses were performed in R (version 4.2.2). A two-sided significance level of 0.05 was selected as the null hypothesis rejection criterion. Linear mixed-effect models were fit using the Ime4^12^ and ImerTest^13^ packages. In multivariable models, all clinically relevant covariates were chosen *a priori*. Before performing inferences on any regression model, residual diagnostics were checked to verify assumptions of normality and homoskedasticity. The responses, AEC and mean IgE, were natural log transformed to induce normality and correct heteroskedasticity. Due to the log transformation, coefficients are interpreted as percent changes rather than absolute changes in the response. Inferences on model coefficients and contrasts were conducted with t-test using the emmeans package^14^ and the F-test.

To assess how log-transformed 12-month mean IgE changed after ETI initiation, we fit a multivariable linear mixed-effect model with a random intercept of patient, regressing over an indicator variable for ETI initiation and adjusting for current age, gender, CFTR mutation, initial disease severity level, *Pseudomonas* spp. colonization, MRSA colonization, other bacterial colonization, fungal colonization, and inhaled steroid use. A linear mixed-effect models was constructed to further investigate the mediating effect of initial disease severity on the ETI treatment effect. The model treated initial disease severity as a factor with four levels.

The relationships between the change in mean IgE after ETI and prior fungal colonization, *Pseudomonas* colonization, or MRSA colonization were each examined individually with two linear mixed-effect models (a univariate and multivariable model with covariates age, gender, CFTR mutation, initial disease severity level, and inhaled steroid use) that had a random intercept for patient. All models included an interaction between the presence of the pathogen and ETI initiation.

To assess how log-transformed AEC changed after ETI initiation, we fit two linear mixed-effect models, a univariate and multivariable model, with a random intercept for patient. While AEC was collected longitudinally, it was still regressed over an indicator variable for ETI initiation because time since initiation was not meaningful. The multivariable model adjusted for age, gender, CFTR mutation, initial disease severity level, *Pseudomonas* spp. colonization, MRSA colonization, other bacterial colonization, fungal colonization, and inhaled steroid use.

## Results

### 1. Baseline Demographics and Characteristics

Summaries of baseline demographics and patient characteristics are presented in Table 1. 108 adult Cystic Fibrosis patients seen at the University of Virginia Adult Cystic Fibrosis Clinic were screened for eligibility, but only 85 unique patients with adequate AEC data, IgE data, or both after exclusion were included in the final analysis. All but five patients with complete AEC data also had complete IgE data. The characteristics of the sample of patients in the IgE analysis were highly similar to the characteristics of the sample of patients in the AEC analysis.

### 2. IgE and ETI Therapy

**Figure 1:**
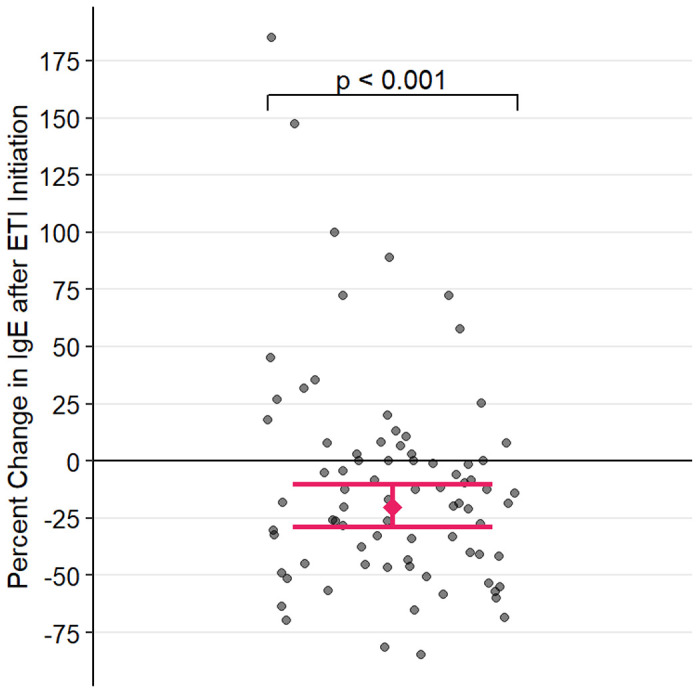
Percent change in mean IgE=Immunoglobulin E after ETI=Elexacaftor/Tezacaftor/Ivacaftor initiation, with 95% confidence intervals computed from the multivariable mixed-effect model. Each point represents the percent change in 12-month mean IgE of a single patient after starting ETI.

IgE data were log-transformed to induce normality prior to analysis. The estimated change in IgE after ETI initiation is plotted in Fig. 1. After adjusting for covariates, patients’ log-transformed 12-month mean IgE decreased by 0.224 (t = −3.838, P < 0.001, 95% CI: [0.108, 0.341]), corresponding to a reduction of 20.10% (95% CI: [10.23%, 28.89%]), following ETI initiation.

### 3. Post-ETI Initiation Change in IgE and Lung Function

After determining that ETI initiation was associated with a change in 12-month mean IgE, we investigated whether initial lung function impacted the magnitude of the change in IgE after ETI. In this mixed model, the reduction of IgE following ETI initiation remained statistically significant (t = 4.060, P < 0.001). However, IgE at baseline did not significantly differ by initial disease severity (F = 1.559, P = 0.206), and the reduction in IgE following ETI therapy did not significantly differ by initial disease severity (F = 0.208, P = 0.890). These results show that in our patients, initial lung function is not associated with the magnitude of change in mean IgE after ETI initiation.

### 4. Post-ETI Change in IgE and Microbiological Colonization

We also investigated whether fungus colonization, *Pseudomonas* colonization, or MRSA colonization alone modified the magnitude of the change in IgE after ETI using subgroup analysis through univariate and multivariable models. Univariate and multivariable models produced similar results, so all values reported below are adjusted estimates.

**Figure 2:**
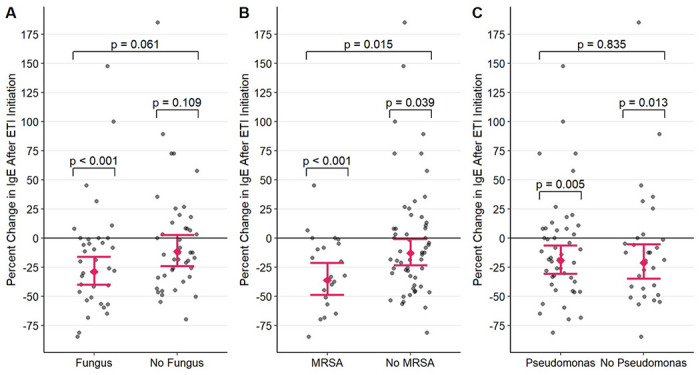
Percent change in mean IgE=Immunoglobulin E after ETI=Elexacaftor/Tezacaftor/Ivacaftor initiation in the (A) presence of fungus or (B) presence of MRSA=*Methicillin Resistant Staphylococcus Aureus* or (C) presence of *Pseudomonas*, with 95% confidence intervals from the multivariable mixed-effect model. Each point represents the percent change in 12-month mean IgE of a single patient after starting ETI. P-values above each group denote the significance of the percent change for that group from 0, and P-values connecting groups denote the significance of the difference in percent change across groups.

As shown in Fig. 2A, after starting ETI, patients with at least one positive fungal culture prior to ETI experienced a significant percent reduction in mean IgE (−28.91%, t = −4.021, P < 0.001, 95% CI: [−39.89%, −15.93%]), but patients with no history of fungal colonization did not (−11.68%, t = −1.622, P = 0.109, 95% CI: [−24.17%, + 2.87%]). The difference in the average magnitude of IgE change between patients with and without at least one positive fungal culture was not significant (t = −1.899, P = 0.061). Figure 2B displays our results for MRSA. The percent reductions in mean IgE following ETI initiation were significant in both patients with (−36.26%, t = −4.176, p < 0.001, 95% CI: [−48.49%, −21.12%]) and without MRSA colonization (−12.83%, t = −2.098, P = 0.039, 95% CI: [−23.48%, −0.69%]). The magnitude of reduction was significantly larger in patients with MRSA colonization (t = 2.481, P = 0.015). Finally, a significant percent reduction in mean IgE was observed both in patients with (−19.32%, t = −2.849, P = 0.005, 95% CI: [−30.48%, −6.36%]) and without (−21.33%, t = −2.544, P = 0.013, 95% CI: [−34.80%, −5.07%]) *Pseudomonas* colonization after starting ETI (Fig. 2C). There was no significant difference in the average magnitude of IgE change between patients with and without *Pseudomonas* colonization (t = 0.209, P = 0.835).

In summary, these results indicate that without considering the effects of the presence of other microorganisms, a reduction in mean IgE was only observed in the presence and not absence of a positive fungal culture and the magnitude of reduction in mean IgE after ETI initiation was only impacted by prior MRSA colonization.

### 5. AEC and ETI

**Figure 3:**
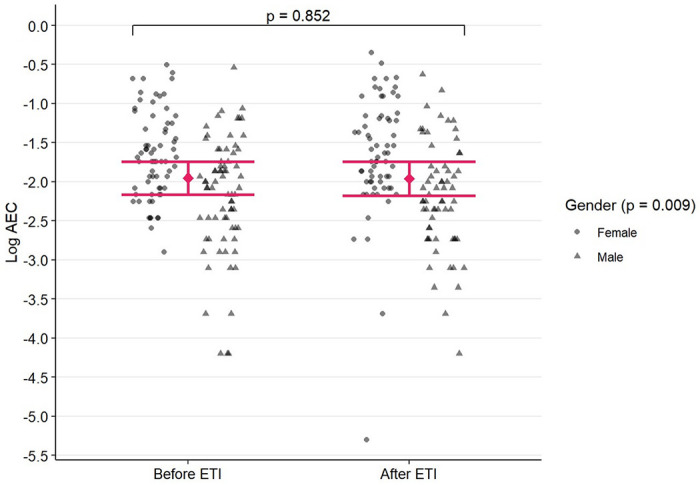
Association between ETI=Elexacaftor/Tezacaftor/Ivacaftor initiation and log AEC=Absolute Eosinophil Count, with 95% confidence intervals computed from the multivariable mixed-effect model. The p-value denotes the significance of change in mean log AEC after starting ETI. Each point represents one observation, and patients can contribute multiple observations. Circles represent observations from female patients, while triangles represent observations from male patients.

AEC data were log-transformed to induce normality prior to analysis. The estimated change in log AEC after ETI initiation is plotted in Fig. 3. Without controlling for any covariates, ETI initiation was not associated with a significant change in log-transformed AEC (t = −0.112, P = 0.911, 95% CI: [−0.144, 0.128]). The multivariable model demonstrated that over the course of the entire study, men have statistically significantly lower AEC than women. On average, men had 37.71% lower AEC than women (t = −2.756, P = 0.009,95% CI: [12.18%, 55.81%]). However, even after adjusting for covariates, ETI initiation still had no association with a change in log-transformed AEC (t = −0.187, P = 0.851, 95% CI: [−0.149, 0.124]).

## Discussion

A large subset of patients with CF develop evidence of T2 inflammation as demonstrated by the presence of blood and airway eosinophilia, robust elevations in their total IgE, and evidence of allergen sensitization.^3–7^ When present, this T2 inflammatory state contributes to the severity of disease and development of disease exacerbations ^6,7^ as can be demonstrated by clinical improvements observed with institution of biologics that target the T2 mediator IL-5. ^8^ T2 inflammation reflects in large part the differentiation of airway epithelial cells as characterized by their production of cytokines – especially the alarmins TSLR IL-25, and IL-33 – that are responsible for immune deviation of Th2 lymphocytes as well as production of chemokines responsible for eosinophil recruitment and activation. ^15,16^ In addition to Th2 lymphocytes, the alarmins drive secretion of the defining T2 cytokines IL-4, IL-5, and IL-33 from numerous other cell types including ILC2s and mast cells. The treatment of patients with CF has been revolutionized by the efficacy of CFTR modulator therapy. The mechanism behind the improvement in patient outcomes is a continued topic of investigation as our understanding of the role of CFTR function evolves. While developing presumably as a complication of the inflammatory state associated with CF, it is less clear whether CFTR dysfunction is directly responsible for T2 inflammation, such as through an impact on the expression of these channels on T and B lymphocytes. And, as such, it is unclear whether institution of CFTR modulator therapy will impact a previously established T2 inflammatory state. To contribute to the growing investigation of the pathogenesis and treatment of CF from an immunologic perspective, this study investigated the impact of CFTR modulation via ETI triple therapy on AEC and IgE as components of the T2 inflammatory signature.

To our surprise, multivariable analysis of AEC data after excluding measurements obtained during exacerbation and/or IV antibiotic use, demonstrated no change following therapy initiation. Eosinophilia is the quintessential defining feature of T2 inflammation.^15,16^ Blood eosinophila, in particular, represents a hematopoietic response to the expression of IL-5. IL-5 is produced by a fairly modest number of cell types including primarily Th2 effector lymphocytes, ILC2s, and mast cells. As such the absence of a change in AEC signifies that initiation of CFTR modulator therapy had no impact on these central defining components of T2 inflammation. This clinically would lead to no impact on the CFAOS presentation and CF exacerbations related to T2 inflammation in PwC and an eosinophilic phenotype.

In contrast to AEC, however, our data demonstrated a ~ 20% reduction in 12-month mean total IgE following ETI triple therapy initiation after controlling for multiple factors including demographic data and inhaled steroid use (Fig. 1). In the absence of an apparent impact on the T2 signature, this impact on total IgE begs an alternative explanation. CFTR dysfunction results in impaired mucus production and clearance, thus promoting microbial colonization in the airway. These microbes and, in particular, fungi, can function as allergens, especially in the presence of a T2^high^ state and it is well recognized that CF patients frequently display aspergillus-specific IgE and, indeed, this disease is often complicated by allergic bronchopulmonary aspergillosis ^17–19^. The CF airway is also frequently contaminated with *Staphylococci*. While not an allergen, *staph* are copious producers of numerous superantigens that non-specifically (that is, in a T cell receptor-independent fashion) can broadly activate numerous families of T lymphocytes. Indeed, CF has been associated with high expression of staph-derived superantigens in the airway^20^. Insofar as many of these T lymphocytes are Th2-like, *staph* superantigens can drive a “storm” of Th2 cytokines, including especially, IL-4 and IL-13, cytokines responsible for IgE production. We would propose that while not directly impacting the T2^high^ state, by improving respiratory function, reducing mucus production, and improving mucociliary clearance CFTR therapy would reduce fungal and *staph* airway colonization. And, as such, through this indirect mechanism, CFTR therapy could reduce IgE concentration. This hypothesis is supported by our data demonstrating a greater reduction in IgE in patients with prior MRSA (Fig. 2). Moreover, our results also demonstrate that IgE was reduced in patients with a history of fungal colonization but was not reduced in patients with no prior history of fungal colonization. IgE. Thus, reduction in antigen (fungal) and superantigen (*Staph*) load secondary to ETI therapy initiation could explain the reduced IgE production in the absence of ETI therapy having an effect on ameliorating the underlying T2^high^ state.

This analysis serves as an important starting point to continue the evaluation of CFTR modulator therapy from an immunologic perspective. These results are intriguing and warrant further studies to elucidate the exact effect of ETI on T2 inflammation. Based on our current findings and previous studies that show a correlation between T2 inflammation based on AEC and IgE levels and frequency of exacerbations^7^, we suspect the ETI may not influence the rate of exacerbation in a subgroup of people with CFAOS and an eosinophilic phenotype. Further studies to elucidate the impacts of possible confounding variables in a larger patient population are warranted. Moreover, studies investigating additional markers of the T2 inflammatory response, especially those utilizing samples obtained from airway will prove valuable.

There are important considerations and limitations regarding this study. There are inherent limitations in performing a retrospective analysis due to limited data measurement frequency, varying frequency of clinical assessment of exacerbation status, and possible unknown microbiological colonization history and allergen sensitization prior to subject care establishment at our center. T2 inflammatory markers are impacted by numerous confounders including exposure to other allergens, systemic and topical corticosteroid use, and others.

## Conclusion

This study demonstrates that there is a statistically significant percent reduction (20.10%, p < 0.001) in mean IgE but no change in AEC following ETI initiation. The lack of change in AEC argues against an impact of ETI therapy on previously established T2 disease. In contrast, we suspect that ETI leads to decreased antigen and superantigen load in the airway as a result of improved mucociliary clearance and it is these changes that drive the decline in IgE. Further studies are warranted to determine the underlying mechanism of ETI impact on T2 inflammation and possible role for asthma immunomodulator therapy post ETI initiation in CFAOS.

## Figures and Tables

**Table 1:** Subject characteristics.

Characteristics	All Patients (n = 85)	IgE Analysis (n = 80)	AEC Analysis (n = 54)
Gender	41 (48.2%)	38 (47.5%)	27 (50.0%)
Male: n (%)	44 (51.8%)	42 (52.5%)	27 (50.0%)
Female: n (%)			

Age	38 (44.7%)	37 (46.3%)	23 (42.6%)
Between 18–30 years	36 (42.4%)	33 (41.2%)	24 (44.4%)
Between 30–50 years	11 (12.9%)	10 (12.5%)	7 (13.0%)
Above 50 years	34.2 (10.7)	33.7 (10.3)	34.7 (11.0)
Mean (SD)	31.8 (12.8)	30.9 (13.0)	32.6 (13.2)
Median (IQR)			

CFTR Mutation Information	49 (57.7%)	48 (60.0%)	32 (59.3%)
delF508 Homozygous	34 (40.0%)	30 (37.5%)	22 (40.7%)
delF508 Heterozygous	2 (2.4%)	2 (2.5%)	0 (0.0%)
Other Mutation			

Initial Disease Severity	23 (27.1%)	22 (27.5%)	13 (24.1%)
Normal (ppFEVI > 90%)	26 (30.6%)	25 (31.2%)	19 (35.2%)
Mild (70% <ppFEV1 ≤ 90%),	25 (29.4%)	23 (28.7%)	17 (31.5%)
Moderate (40% <ppFEV1 ≤ 70%)	10 (11.8%)	10 (12.5%)	4 (7.4%)
Severe (ppFEVI ≤ 40%)	1 (1.2%)	0 (0.0%)	1 (1.9%)
Not Available			

Pre-ETI Inhaled Steroids	51 (60.0%)	49 (61.3%)	30 (55.6%)
Yes	33 (38.8%)	30 (37.5%)	24 (44.4%)
No	1 (1.2%)	1 (1.3%)	0 (0.0%)
Not Available			

Colonization	25 (29.4%)	21 (26.3%)	17 (31.5%)
MRSA	50 (58.8%)	48 (60.0%)	29 (53.7%)
*Pseudomonas*	32 (37.6%)	31 (38.8%)	21 (38.9%)
Other Bacteria	36 (42.4%)	36 (45.0%)	25 (46.3%)
Fungus	1 (1.2%)	1 (1.3%)	0 (0.0%)
Not Available			

Note: “All patients” refers to all patients included either the IgE analysis, AEC analysis, or both.

IgE = Immunoglobulin E, ETI = Elexacaftor/Tezacaftor/Ivacaftor, ppFEV1 = percent predicted Forced Expiratory Volume in 1 second, MRSA = *Methicillin Resistant Staphylococcus Aureus*, AEC = Absolute Eosinophil Count

## Data Availability

The datasets generated and/or analysed during the current study are not publicly available due to possibly leading to subject identification, (single center, and rare disease subjects) but the statistical analysis is available from the corresponding author on reasonable request.

## Abbreviations

AEC: Absolute Eosinophil Count
CF: Cystic Fibrosis
CFAOS: CF-asthma overlap syndrome
CFTR: Cystic Fibrosis Transmembrane Regulator
ETI: Elexacaftor/Tezacaftor/Ivacaftor
IgE: Immunoglobulin E
IL: interleukin
TSLP: Thymic stromal lymphoproietin
ppFEV1: percent predicted Forced Expiratory Volume in 1 second
MRSA: Methicillin Resistant Staphylococcus Aureus
